# Unveiling the enigma from sick to beauty: Hungry to standardize metrics for dynamic cerebral autoregulation

**DOI:** 10.1113/EP091781

**Published:** 2024-02-25

**Authors:** Michael M. Tymko

**Affiliations:** ^1^ Integrative Cerebrovascular and Environmental Physiology SB Laboratory, Department of Human Health and Nutritional Sciences, College of Biological Science University of Guelph Guelph Ontario Canada

**Keywords:** cerebral autoregulation, cerebral blood flow, transcranial Doppler ultrasound

1

In this issue of *Experimental Physiology*, Olsen et al. (2024) present a pivotal article addressing a pervasive challenge in both basic and clinical physiology. Their review paper, titled ‘Myths and methodologies: Evaluation of dynamic cerebral autoregulation via the mean flow index’, sends a cautionary note challenging the long‐term decadence of mean velocity index‐based measures in evaluating dynamic cerebral autoregulation (dCA) due to methodological inconsistencies, resulting in compromised validity and reproducibility. Additionally, Olsen et al. (2024) advocate for the enhancement of experimental standardization, something that we should strive for and promote across all facets of human physiology. The authors use this opportunity to highlight their open‐source software, ‘clintools’, in the hope of improving the overall quality of data in the cerebral autoregulation (CA) field.

The mean velocity index (Mx)‐based measure of dCA characterized by Czosnyka et al. (1996) showed tremendous utility in their prospective dataset collected at the internationally renowned Addenbrooke's Hospital. The authors demonstrated that Mx was related to patient outcome in individuals with severe brain injury. However, since the inception of this methodological approach, like with many others, there have been methodological caveats and challenges with its implementation, elegantly summarized throughout the review by Olsen et al. (2024). For example, this approach uses linear modelling despite CA emerging as a non‐linear mechanism that dynamically interacts with various physiological processes (see examples in the following paragraph). To navigate these complexities, ongoing analytical innovations are essential, enabling the multivariate quantification of both linear and non‐linear properties of cerebral circulation. Mx leverages spontaneous fluctuations in physiological parameters, including Mayer waves and respiratory‐induced alterations in arterial blood pressure, providing valuable insights into an individual's CA capacity during undisturbed resting periods. This attribute makes this approach particularly useful in clinical environments. Nonetheless, the relatively modest amplitude of these variations, leading to a diminished signal‐to‐noise ratio, could potentially undermine the reliability of Mx and other similar approaches (e.g., PRx) (Brassard et al., 2023).

Cerebral autoregulation also exhibits ‘non‐stationary’ behaviour, indicating that it is not constant over time, thereby impacting the reproducibility of metrics derived from spontaneous fluctuations (Panerai, 2014). It is possible that CA could be different on an hour‐to‐hour, and maybe even minute‐to‐minute basis! This variability could stem from significant measurement errors or, more likely, from inherent physiological fluctuations arising from changes in arterial partial pressure of CO_2_, O_2_, sympathetic and parasympathetic nerve activity, metabolism, body temperature, sex hormones, endothelial function, intracranial pressure, intrathoracic pressure and blood rheology (Tzeng & Ainslie, 2014; Willie et al., 2014). To compound the complexity of dCA even further, another significant issue highlighted in Olsen and colleagues' review is the lack of convergence among commonly applied dCA metrics (e.g., rate of regulation, transfer function, autoregulation index). This raises doubts about the precise interpretation of dCA and complicates the selection of an appropriate index as dCA continues to puzzle physiologists. A real enigma you might say!

Like many dCA metrics, it relies on continuous measurements of cerebral blood flow (or velocity). The predominant method for dCA assessment involves transcranial Doppler ultrasound, which assumes constant blood vessel diameter, thereby using blood velocity as a proxy for blood flow. However, alterations in vascular diameter can lead to errors in flow estimation. It is challenging to dismiss the possibility of diameter changes in clinical cohorts undergoing medication regimens and lifestyle adjustments. While such alterations in diameter may not be common in humans at the level of the intracranial conduit vessels (i.e., middle cerebral and posterior cerebral arteries), where velocity recordings are typically made, sudden hypotension can trigger passive reductions in vascular diameter of up to 10% of the baseline value (Kontos et al., 1978). Importantly, even a minor change in diameter, which might not be easily detected by cerebral magnetic resonance imaging, could negate observed changes in calculated vascular resistance shortly after a blood pressure fluctuation (Kontos, 1989), significantly impacting the reliability of dCA metrics. Hence, researchers utilizing transcranial Doppler must meticulously account for the impact of potential experimental interventions on the calibre of insonated vessels. Remarkable progress in transcranial colour Doppler ultrasound of intracranial vessels and Duplex ultrasound of extracranial vessels provides the ability to gauge both blood velocity and vessel diameter, enabling precise blood flow computations. These advancing technologies, coupled with software that facilitates real‐time evaluation of blood velocity and vessel diameter measurements, represent promising bedside instruments on the horizon!

This important review by Olsen et al. (2024) critically examined the strengths and limitations of Mx as an index of dCA. The authors highlight methodological inconsistencies that compromise the validity and reproducibility of Mx, advocating for enhanced experimental standardization—an important and translatable message to all physiologists.

## AUTHOR CONTRIBUTIONS

Sole author.

## CONFLICT OF INTEREST

The author declares no conflicts of interest.

## FUNDING INFORMATION

No funding was received for this work.
